# Cricket, commerce, and public health: promotion of tobacco, alcohol, and high in fat, sugar, and salt products

**DOI:** 10.3389/fdgth.2025.1503680

**Published:** 2025-06-26

**Authors:** Prashant Kumar Singh, Rupal Jain, Vandana Tamrakar, Sanchita Roy Pradhan, Sagarika Rout, Chandresh Pragya Verma, Amit Yadav, Upendra Bhojani, Yatan Pal Singh Balhara, Shalini Singh

**Affiliations:** ^1^ Division of Preventive Oncology & Population Health, WHO FCTC Knowledge Hub on Smokeless Tobacco, ICMR–National Institute of Cancer Prevention and Research, Noida, India; ^2^ Faculty of Medical Research, The Academy of Scientific and Innovative Research (AcSIR), New Delhi, India; ^3^ School of Public Policy, Indian Institute of Technology, New Delhi, India; ^4^ Vital Strategies, New Delhi, India; ^5^ Institute of Public Health, Bengaluru, India; ^6^ All India Institute of Medical Sciences (AIIMS), New Delhi, India

**Keywords:** tobacco promotion, alcohol advertising, ultra-processed foods, surrogate advertisements, over-the-top (OTT) platforms, public health regulation

## Abstract

**Background:**

Increasing incidences of non-communicable diseases globally present a major public health challenge, with tobacco, alcohol, and ultra processed food products high in fat, sugar, and salt (HFSS) contributing significantly to this epidemic. Despite regulatory efforts, loopholes persist, allowing companies to promote such products through surrogate advertisements and new media platforms. This study investigates advertisements aired during the Men's Cricket World Cup 2023 on the Over-the-Top (OTT) platform.

**Methods:**

A comprehensive analysis of advertisements aired during the World Cup matches on OTT platform during October-November 2023 was undertaken to assess the extent and type of advertising of alcohol, tobacco and HFSS products. A standardized observation protocol was followed, documenting the frequency, type, and celebrity featured in each advertisement. The observed advertisements were categorized into six segments including surrogate tobacco and alcohol, soft drinks, energy drinks, edible products commonly consumed by children, and other edibles/beverages.

**Results:**

Observations show that 80.9% (*n* = 1,769) of total advertisements promoted tobacco, alcohol and HFSS products. Notably, surrogate tobacco advertisements were predominantly displayed during matches involving the Indian team, accounting for 86.7% of the total surrogate tobacco advertisements. Edible products commonly consumed by children comprised 60.6% of unhealthy advertisements during over-breaks. Celebrity endorsements, particularly by Bollywood actors and Indian cricketers were common.

**Conclusion:**

Observations reveal a concerning prevalence of advertisements promoting tobacco, alcohol, and HFSS products. Children emerged as a particularly vulnerable target for advertisement-induced consumption behaviors. These findings highlight the need for stricter regulations and effective enforcement to curb the promotion of unhealthy products.

## Introduction

The global burden of non-communicable diseases (NCDs) continues to escalate, with NCDs responsible for 41 million deaths annually, accounting for 74% of all global mortality (1). The leading causes include cardiovascular diseases, cancers, respiratory diseases, and diabetes (2). The World Health Organization (WHO) has identified tobacco, alcohol, unhealthy dietary products, and sugar-sweetened beverages (SSBs) among others as key risk factors for NCDs (3). Consequently, recognizing the significant influence of advertisements on consumer behaviour and their massive exposure in today's world, the WHO has recommended limiting the marketing exposure of unhealthy foods, especially to children (4–6) as well as underscored the importance of prohibiting direct and indirect tobacco advertisements globally under Article 13 of the World Health Organization Framework Convention on Tobacco Control (7).

While these regulations exist, they were primarily developed in the context of traditional media. With the rapid shift to digital platforms, there is growing evidence that companies are now turning to online streaming services and influencer-driven campaigns to reach larger and younger audiences. This form of marketing is especially concerning given its subtlety and pervasive nature, often escaping regulatory oversight. The promotion of unhealthy products through sporting events has been a commonly used strategy by industry to promote their products among all age-sex categories (8). Evidence from high-income countries documents instances of promotion of tobacco, alcohol and sugar-sweetened beverages in Olympic Games, men's FIFA World Cup, tennis tournaments and others (8). However, similar systematic evidence is very limited from low-and-middle income countries, particularly countries like India which houses the largest population on earth and an ever growing consumer market.

In India, the Food Safety and Standard Act 2006 prohibits misleading food advertisements (9). However, there is no direct prohibition on advertisements of HFSS products in India yet, leaving a regulatory gap that allows the promotion of these products (10, 11). Further, legislative measures such as the Cigarettes and Other Tobacco Products Act (COTPA), 2003, under Section 5 prohibits any direct and indirect advertisement of tobacco products (12). Any direct and indirect alcohol advertisement has also been banned by the Government of India through the Cable Television Network (Regulation) Act, 1995 and the Advertising Standards Council of India under Ministry of Information and Broadcasting (13). Overall, the recent consumer protection law and the guidelines issued thereunder prohibit surrogate advertising of products which are prohibited from being advertised under any law in force or under any rules or regulations made thereunder (14). Despite these regulatory efforts, alcohol, tobacco and food companies persist in promoting their brands and products through surrogate and misleading advertisements, evading existing restrictions (15).

This regulatory gap is especially concerning given the massive viewership of sports events on these platforms. For instance, the ICC Men's Cricket World Cup 2023 recorded over one trillion viewing minutes globally and 422 million viewers in India alone, via Disney Star Network. The final match between India and Australia peaked at 87.6 billion live viewing minutes globally, making it the most-watched ICC match ever.

Given the growing viewership of sports events and the increasing marketing efforts by the alcohol, tobacco, and HFSS industries, our study hypothesized a high prevalence (>50%) of such advertisements during the ICC Men's Cricket World Cup 2023, particularly during matches involving the Indian team. Thus, this study aims to investigate the volume and nature of advertisements related to alcohol, tobacco, and HFSS products during the ICC Men's Cricket World Cup 2023 as streamed on a major OTT platform, with a focus on their frequency, target audience, product type, and use of celebrity endorsements.

The rest of the article is organized as follows: First we briefly discuss the methodology adapted. Next, we present our results showing the prevalence of unhealthy product advertisements. Following this, we discuss the implications of our findings. Finally, we conclude with policy recommendations and highlight limitations along with future research directions.

## Methods

The ICC Men's Cricket World Cup 2023, hosted in India during October and November 2023, gained global attention and unprecedented viewership. The tournament lasted for 46 days in which 10 countries (Afghanistan, Australia, Bangladesh, England, India, the Netherlands, New Zealand, Pakistan, South Africa and Sri Lanka) participated.

A comprehensive analysis of advertisements aired during the ICC Men's Cricket World Cup matches on OTT platform from 5th October 2023 - 19th November 2023 was undertaken to assess the extent and type of advertising of alcohol, tobacco and HFSS products. A spreadsheet using Microsoft Excel (version 2010) was used for manual data entry and Stata version 14.1 was used for analysis. Four trained researchers from ICMR-National Institute of Cancer Prevention and Research independently recorded the data for cross-verification, to ensure rigor and reduce biases. In cases of inconsistency among the four trained researchers during data categorization, it was resolved through consensus discussions.

A standardized observation protocol was followed, documenting the frequency, type, and celebrity featured in each advertisement. The observed advertisements were categorized into six segments including surrogate tobacco, surrogate alcohol, soft drinks, energy drinks, edible products commonly consumed by children, and other edibles/beverages. The soft drinks and energy drinks were categorized under HFSS due to their high sugar content. HFSS products commonly consumed by children were allotted a separate category given their significant impact on children's dietary habits. These products included, but were not limited to, chocolates, potato chips, noodles, and biscuits.

The frequency of advertisements was analyzed and categorized based on the countries playing the matches being observed and the exhibition categories of advertisements, such as display during Over Breaks (the delivery of six consecutive legal balls by one bowler), display at the bottom of the screen along with live scores, and On-field (As seen on OTT) advertising. For an advertisement to be included in our coding, the product or brand logo had to be visibly identifiable for a minimum of two seconds. Only distinct advertisement occurrences were counted.

## Results

### Promotion of alcohol, tobacco and HFSS products in ICC men's cricket world cup 2023

The total streaming time for all live matches on the OTT platform was approximately 341 h, with an average streaming time of 7 h and 10 min per match. The results reveal a high percentage of advertisements promoting unhealthy products, totalling 1,769 (80.9%) instances out of 2,118 (Table 1). Among all matches with different countries, matches where the host country (India) was playing had the highest share of such unhealthy advertisements (44.8%, *n* = 792), including during the breaks between overs (48.6%, *n* = 426), bottom of the screen (49.6%, *n* = 171) and on-field (35.6%, *n* = 195) advertising.

**Table 1:** Unhealthy products advertisement during ICC men's world Cup 2023 on OTT platform.

Product types	Advertisement instances [%(n)]								
	Total	Indian team matches		Asian teams matches (other than India)		Non-Asian team matches		Asian & non-Asian team matches	
Display During Over breaks (the delivery of six consecutive legal balls by one bowler)								
*n*	%	*n*	%	*n*	%	*n*	%	*n*
Surrogate tobacco	45	86.7	39	0.0	0	0.0	0	13.3	6
Surrogate alcohol	50	28.0	14	6.0	3	34.0	17	32.0	16
Soft drinks	186	18.8	35	11.8	22	18.8	35	50.5	94
Energy drinks	64	62.5	40	7.8	5	20.3	13	9.4	6
Edible products commonly consumed by children	531	56.1	298	4.9	26	11.3	60	27.7	147
Other Edibles/Beverages	108	12.0	13	9.3	10	41.7	45	37.4	40
^a^Others	124	18.6	23	11.3	14	27.4	34	42.7	53
Total advertisements (A)	1,108	41.7	462	7.2	80	18.4	204	32.7	362
^b^Total unhealthy products advertised (A^1^)	876	48.6	426	6.4	56	14.3	125	30.7	269
	Display at the bottom of the screen along with live scores								
Surrogate tobacco	0	0.0	0	0.0	0	0.0	0	0.0	0
Surrogate alcohol	114	48.3	55	4.4	5	14.0	16	33.3	38
Soft drinks	106	43.4	46	13.2	14	16.0	17	27.5	29
Energy drinks	28	96.4	27	0.0	0	0.0	0	3.6	1
Edible products commonly consumed by children	95	45.3	43	4.2	4	20.0	19	30.5	29
Other Edibles/Beverages	3	0.0	0	33.4	1	66.7	2	0.0	0
^a^Others	116	38.5	45	11.2	13	11.2	13	38.7	45
Total advertisements (B)	462	46.8	216	9.1	42	14.5	67	29.7	137
^b^Total unhealthy products advertised (B^1^)	345	49.6	171	8.1	28	15.4	53	27.0	93
	Display in the In-field (As seen on OTT)								
Surrogate Alcohol	548	35.6	195	11.5	63	18.4	101	34.5	189
Total unhealthy products advertisement (C^1^)	548	35.6	195	11.5	63	18.4	101	34.5	189
Total (A + B + C)	2,118	41.2	873	8.7	185	17.6	372	32.5	688
Total (A^1^ + B^1^ + C^1^)	1,769	44.8	792	8.3	147	15.8	279	31.2	551
Number of matches	48	11		5		11		21	
Streaming time	341 h	75 h, 28 min		34 h, 40 min		77 h, 17 min		148 h, 17 min	
Duration of Matches	7 h, 10 min	6 h, 8 min		6 h, 8 min		7 h, 17 min		7 h, 22 min	
Average live viewership (Disney + Hostar)	4.4 million viewers	26 million viewers		13 million viewers		3.8 million viewers		5.3 million viewers	

^a^ Others: Advertisement of edibles or edible brands that does not fall into either of the categories (such as Salt, Turmeric, Nutraceuticals, etc).

^b^ Total unhealthy products includes tobacco, alcohol, soft drinks, energy drinks, and edible products commonly targeted towards children.

Particularly for India, 90.72% (*n* = 792) of the total advertisements belonged to the unhealthy products category, which includes tobacco, alcohol, soft drinks, energy drinks, and edible products commonly consumed by children. One of the most notable patterns observed was that surrogate smokeless tobacco (SLT) advertisements were aired maximum at 86.7% during the breaks between overs when India was playing. This trend was notably absent or negligible during matches played by other countries.

Advertisements of HFSS products commonly consumed by children occupied the highest frequency among various categories of advertisements broadcasted during breaks (60.6%; *n* = 531).

### Endorsements by bollywood and cricket celebrities

A higher percentage of advertisements were broadcasted without any celebrity endorsements during breaks between overs (65.5%, *n* = 574) and bottom of the screen display (64.0%, *n* = 219). However, when endorsements did occur, Bollywood (17.5%, *n* = 153) and Cricket (17.0%, *n* = 149) celebrities were seen equally endorsing the unhealthy products during the breaks between overs (Table 2). The celebrity cricketers were in the forefront for the unhealthy product advertisements on display panel (bottom of the screen along with live scores) (27.4%, *n* = 94).

**Table 2:** Proportion of [n (%)] unhealthy products advertisement and their endorsement by celebrities during ICC men's world Cup 2023 on OTT platform.

Product types	Advertisement instances [Proportion, *n* (%)]						
	Bollywood celebrity			Cricketers celebrity No celebrity			
Display during over breaks^a^						
Total	%	*n*	%	*n*	%	*n*
Surrogate tobacco	45	46.7	21	35.6	16	17.8	8
Surrogate alcohol	50	34.0	17	0.0	0	66.0	33
Soft drinks	186	49.5	92	0.0	0	50.5	94
Energy drinks	64	6.3	4	1.6	1	92.2	59
Edible products commonly consumed by children	531	3.6	19	24.9	132	71.6	380
Other Edibles/Beverage	108	13.0	14	0.9	1	86.1	93
Others^b^	124	0.0	0	88.7	110	0.0	0
Total advertisements	1,108	15.1	167	23.5	260	60.2	667
Total unhealthy products advertisements	876	17.5	153	17.0	149	65.5	574
	Display at the bottom of the screen along with live scores						
Surrogate Tobacco	0	0	0	0	0	0	0
Surrogate Alcohol	114	26.3	21	33.3	38	40.4	46
Soft Drinks	106	0.0	0	0.0	0	100.0	106
Energy drinks	28	0.0	0	85.7	24	14.3	4
Edible products commonly consumed by children	95	0.0	32	33.7	32	66.3	63
Other Edibles/Beverage Others^b^	3	0.0	0	0.0	0	100.0	3
Total advertisements	116	0.0	53	87.9	102	12.1	14
Total unhealthy products advertisements	462	6.5	53	42.4	196	51.1	236

^a^ Over breaks: the delivery of six consecutive legal balls by one bowler.

^b^ Others: Advertisement of edibles or edible brands that does not fall into either of the categories (such as Salt, Turmeric, Nutraceuticals etc).

## Discussion

This study provides the first evidence from low-and-middle income countries showing the extent of promotion of unhealthy products during global sports events like ICC's Men's Cricket World Cup held in 2023 in India. This poses challenges for public health with respect to growing burden of NCDs associated morbidity and mortality which are avoidable. The study results underscore the need for action by policy-makers to mitigate the impact of commercial interests, amplified through such promotions during sports events, in the larger interest of public health.

Enforcing bans or comprehensive restrictions on alcohol advertising, sponsorship, and promotion has been identified as one of the five key interventions by the WHO to address problems due to alcohol use (16). However, the adherence to these guidelines by OTT platforms appears to be poor. The public health measures for modifying behavioural risk factors should keep pace with advancing communication and media technology such as OTT platforms that are being used for promoting unhealthy products.

Globally, strategies used by the tobacco industry to circumvent regulation are being adopted by the food industry. These include influencing policy makers, spreading misinformation based on poorly conducted research, recruiting social media influencers and placing advertisements of HFSS products in films, TV programmes and online streaming content (8).

The regulatory framework to control promotion of unhealthy products varies widely across the globe. For example, London revised its Transport for London (TfL) advertising policy in 2019, prohibiting advertisement of food or non-alcoholic drinks high in fat, salt, and/or sugar on services run or regulated by TfL. Similarly, Amsterdam banned billboard advertisements in 2018 for unhealthy products targeting children and teenagers in metro stations (17).

This study shows that the advertisement industry has shifted from traditional media to platforms like OTT in India as well. These platforms continue to display a high volume of advertisements for HFSS products, indicating a gap in enforcement. Children have been recognized as being very vulnerable to behaviours induced by advertisements (18). This susceptibility has been frequently exploited by industry, with the highest instances of edible products commonly consumed by children. Such endorsements also undermine government efforts to raise awareness among children about the dangers of tobacco, alcohol, and unhealthy diet.

The Independent High-Level Commission on NCDs setup by WHO recommended governments to engage constructively with private industries (except tobacco industry), including leisure and sports industries to promote physical activity and give priority to restricting marketing of unhealthy products to children (19, 20).

### Implications

This study contributes to the growing body of literature on commercial determinants of health by providing empirical evidence from a major sporting event in a low-and-middle income country context (24). It extends previous research predominantly conducted in high-income countries by demonstrating similar patterns of unhealthy product promotion in the Indian market. The study also advances understanding of how digital platforms create new avenues for circumventing traditional advertising regulations.

The findings have several important implications for policy development. First, there is an urgent need to update existing regulatory frameworks to encompass digital and OTT platforms, which currently operate in a regulatory grey area (21, 22). Second, enforcement mechanisms need strengthening, particularly for surrogate advertising that cleverly evades direct advertising bans. Third, specific protections for children are essential, given their vulnerability to advertisement-induced behaviors.

Policy-makers should consider implementing comprehensive advertising restrictions similar to those adopted in London and Amsterdam, adapted to the Indian context. Additionally, stricter monitoring mechanisms for OTT platforms are needed, along with clear consequences for non-compliance.

For consumers, particularly parents, these findings highlight the importance of media literacy and critical evaluation of advertising content. Understanding the persuasive intent behind celebrity endorsements can help in making more informed choices. Educational campaigns about the health risks associated with tobacco, alcohol, and HFSS products are crucial for building consumer awareness.

### Limitations and future research

This study has several limitations that should be acknowledged. The analysis focused on a single OTT platform, which may not fully capture the range of advertisements across other platforms. The categorization of product types relied on researcher interpretation, which, although cross-checked for consistency, may involve some degree of subjectivity. Brand-level analysis was intentionally omitted due to the scope focusing on product categories rather than brand-specific marketing.

Future research should examine multiple OTT platforms simultaneously to provide a more comprehensive view of advertising practices. Studies assessing the actual impact of these advertisements on consumer behavior would be valuable. Cross-country comparative studies could help understand how different regulatory environments affect advertising practices. Additionally, longitudinal studies tracking changes in advertising patterns following policy interventions would provide insights into regulatory effectiveness.

Research examining the effectiveness of different intervention strategies in reducing unhealthy product advertising would inform evidence-based policy development. Studies focusing on children's responses to celebrity endorsements in advertisements could guide targeted protective measures.

## Conclusion

This study reveals a concerning prevalence of advertisements promoting tobacco, alcohol, and HFSS products during the ICC Men's Cricket World Cup 2023, with 80.9% of all advertisements falling into these categories. Children emerged as particularly vulnerable targets, with edible products commonly consumed by them comprising the highest frequency of unhealthy advertisements during over-breaks (23).

There is an urgent need for governments, national and international sports federations to develop policies restricting entertainment industries from promoting unhealthy products during sports events and their broadcasting globally. As the landscape of entertainment/sports media continues to evolve, particularly with the rise of digital and OTT platforms, protocols to regulate the industry should be updated, revised and made future-ready.

These measures are crucial not only for protecting vulnerable populations, especially children and adolescents, but also for aligning commercial practices of the entertainment industry with broader public health goals. Stronger enforcement mechanisms, regular monitoring, and accountability measures will be key to ensuring that commercial interests do not override public health priorities.

The findings underscore that despite numerous laws and regulations, there remains a significant gap between policy intention and implementation. Addressing this gap is essential for India to achieve its aim of reducing premature deaths from NCDs by 25% by 2025.

## Data Availability

The original contributions presented in the study are included in the article/Supplementary Material, further inquiries can be directed to the corresponding author.
